# α‐Synuclein Forms Distinct Micelle‐Like Assemblies at Low Ionic Strengths

**DOI:** 10.1002/advs.77300

**Published:** 2026-08-21

**Authors:** Sophie Hertel, Soumik Ray, Federica Saraceno, Giacomo Nisini, Georgia Nasi, Thomas Oliver Mason, Antonin Kunka, Effrosyni Agrimaki, Dannie Juul Olesen, Francesco Simone Ruggeri, Alexander K. Buell

**Affiliations:** ^1^ Department of Biotechnology and Biomedicine Technical University of Denmark, Kgs Lyngby Denmark; ^2^ Physical Chemistry and Soft Matter Wageningen University and Research Wageningen The Netherlands; ^3^ Laboratory of Organic Chemistry Wageningen University and Research Wageningen The Netherlands

## Abstract

Intrinsically disordered proteins (IDPs) often associate into biomolecular condensates. While micron‐sized condensates are observed above a well‐defined saturation concentration, the formation of nanoscale clusters also occurs at subsaturated concentrations. The diversity and physical nature of such nanoscale clusters remain underexplored with respect to the well‐established condensate state. Here we show that the Parkinson's disease‐associated protein α‐synuclein forms highly monodisperse assemblies at low ionic strength, distinct from the larger polydisperse assemblies previously observed at higher ionic strengths. Using a range of biophysical methods, we identify monodisperse clusters of α‐synuclein composed of ∼20 molecules that maintain their size over a broad concentration range. These clusters are stable over time and do not progress to amyloid fibril formation. Their response to mutational perturbations and chemical destabilization, as well as their thermal cycling support a micelle‐like assembly. Increasing ionic strength shifts the system toward the formation of larger, polydisperse nanoclusters and condensates that promote amyloid fibril formation. Our results reveal a hitherto uncharacterized assembly state of α‐synuclein and highlight the complexity of nanoscale assembly processes by IDPs.

## Graphical abstract

At high ionic strength, α‐synuclein forms diverse assemblies, including oligomers, fibrils, and condensates. Here, we show that at low ionic strength, α‐synuclein adopts a distinct, low‐abundance assembly state. These assemblies maintain a constant size above a critical concentration and do not coalesce, suggesting a micelle‐like organization. Mutational, chemical, and thermal analyses support this model, revealing a previously unrecognized α‐synuclein assembly state.

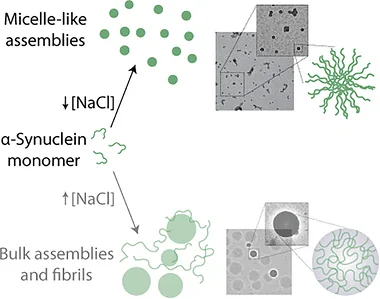

## 1 Introduction

Self‐assembly and self‐association pathways of intrinsically disordered proteins (IDPs) such as TDP‐43, tau, amyloid‐β, and α‐synuclein (α‐Syn) have been studied extensively over the last decades [1, 2, 3, 4]. These proteins can form oligomers with various degrees of secondary structure that are toxic to cells as well as amyloid fibrils, and these assembled states are key pathological features of neurodegenerative disorders such as Frontotemporal dementia, Alzheimer's, and Parkinson's disease [5, 6]. The conversion of monomers into amyloid fibrils can follow multiple pathways. Each pathway involves a series of intermediate states that can have very different lifetimes [6]. In in vitro studies with purified individual proteins, many amyloid‐forming proteins can undergo liquid‐liquid phase separation (LLPS), forming µm sized concentrated droplets suspended in a dilute background solution [7, 8, 9, 10]. The surfaces of such concentrated dense phase droplets can serve as effective nucleation sites for fibrils [11, 12, 13]. Many proteins that undergo LLPS in purified form in vitro are also found in biomolecular condensates and/or membrane‐less organelles in cells [14, 15, 16]. Based on these observations, biomolecular condensates have been widely considered as possible starting points for fibril formation in vivo, even though this view has been challenged in recent studies involving condensates with complex compositions and multiple components [17].

Associative polymers such as proteins can undergo a percolation transition that leads to the formation of system‐spanning networks of clusters, or macroscopic phase separation at a saturation/critical concentration (C_crit_) [18, 19]. Depending on the relative positions of the percolation threshold (C_perc_) and the C_crit_, proteins may form nanoscale clusters, network‐like assemblies, or bulk condensates [20]. Considering the confined volumes within living cells, recent years saw an increased focus of the research community on probing assemblies of phase separating proteins at the nanoscale. By using approaches such as small angle X‐ray scattering, nanoparticle tracking analysis, and fluorescence correlation spectroscopy, nanoscale clustering at sub‐saturated concentrations, i.e., in the absence of optically visible condensates, has been observed for hnRNPA1 [21], FUS [22, 23], members of the FET protein family [19], α‐Syn [24] and TDP‐43 [25, 26], respectively. In some cases, these nanoclusters are speculated to be intermediates that precede the formation of larger, macroscopic condensates and, eventually, amyloid fibrils.

One protein system that features sub‐saturated nanoclusters, acting as temporal pre‐cursors for condensates, is the Parkinson's associated protein α‐Syn [24]. α‐Syn shows features of a triblock copolymer comprised of an N‐terminal amphipathic domain, a hydrophobic core (NAC) and an acidic C‐terminal domain. This unique domain architecture enables α‐Syn to adapt a wide range of assembly states, which can be on‐ or off‐pathway to fibril formation. These include stable or transient oligomers [27, 28, 29], macroscopic condensates [8], hydrogels [30], and, as recently demonstrated by our group, nanoclusters [24] (Table S1). The formation of these nanoclusters, similarly to α‐Syn oligomers and amyloid fibrils, is promoted when electrostatic interactions are screened by the addition of salt [31, 32].

Here, we demonstrate the existence of a previously unknown solution‐phase assembly state of α‐Syn, occurring in conditions of low ionic strength. Using an array of biophysical techniques including mass photometry (MP), transmission electron microscopy (TEM), atomic force microscopy (AFM), dynamic light scattering (DLS), and flow‐induced dispersion analysis (FIDA), we find that in the absence of added salt, α‐Syn adopts a distinct assembly state composed of ∼20 monomers, at low population. We show that these assemblies are monodisperse, stabilized in size above a critical concentration, and do not coalesce, suggesting a micelle‐like organization. By tuning electrostatic interactions and attractive forces, α‐Syn can shift from this micelle‐like state to larger nanoclusters or condensates of less well‐defined sizes, revealing a sensitive balance between electrostatic and hydrophobic interactions in shaping its assembly behavior. Furthermore, we also demonstrate through the study of Ddx4N1 and the C‐terminal low‐complexity domain of TDP‐43 (TDP43‐LCD) that while the nanoscale assembly state of condensate‐forming proteins is widespread, the formation of micelle‐like assembly states, if accessible at all to any given protein, is associated with very specific sets of solution conditions.

## 2 Results

### 2.1 Spontaneous Assembly of α‐Syn at Low Ionic Strengths

We previously showed that electrostatic screening of the negative net charge of α‐Syn, which is mostly concentrated on the C‐terminus, promotes the formation of both nanoclusters and macroscopic condensates [24]. Therefore, we expected that under low ionic strength conditions, the critical concentration (C_crit_) of such assembly states would shift to much higher protein concentrations. Surprisingly, in MP measurements, we observed higher‐order assemblies even in the absence of NaCl (Figure 1a), distinct from monomeric α‐Syn, which with a molecular weight of 15 kDa lies below the detection limit of our MP instrument (∼40 kDa), and which would therefore not be observed. Interestingly, the C_crit_ of these assemblies was close to 1 µM, which was at least an order of magnitude lower than the C_crit_ for high‐salt nanocluster [24] formation and two orders of magnitude lower than macroscopic condensate formation [8, 33]. Furthermore, the apparent mass (MW_app_) of the clusters stabilized around ∼300–400 kDa (∼20–26 α‐Syn molecules) at concentrations ≥10 µm and did not increase in size even with significantly increasing protein concentration (Figure 1a, left). Addition of 5% (w/v) PEG‐8000, a molecular crowder used to induce the formation of high‐salt nanoclusters and condensates [8, 24, 33], did not change the stable MW_app_ (Figure 1a, right). Throughout the manuscript, we will refer to these structures as low‐salt assemblies (LSAs) to distinguish the nomenclature from other assembly states of α‐Syn described in previous studies.

**FIGURE 1:**
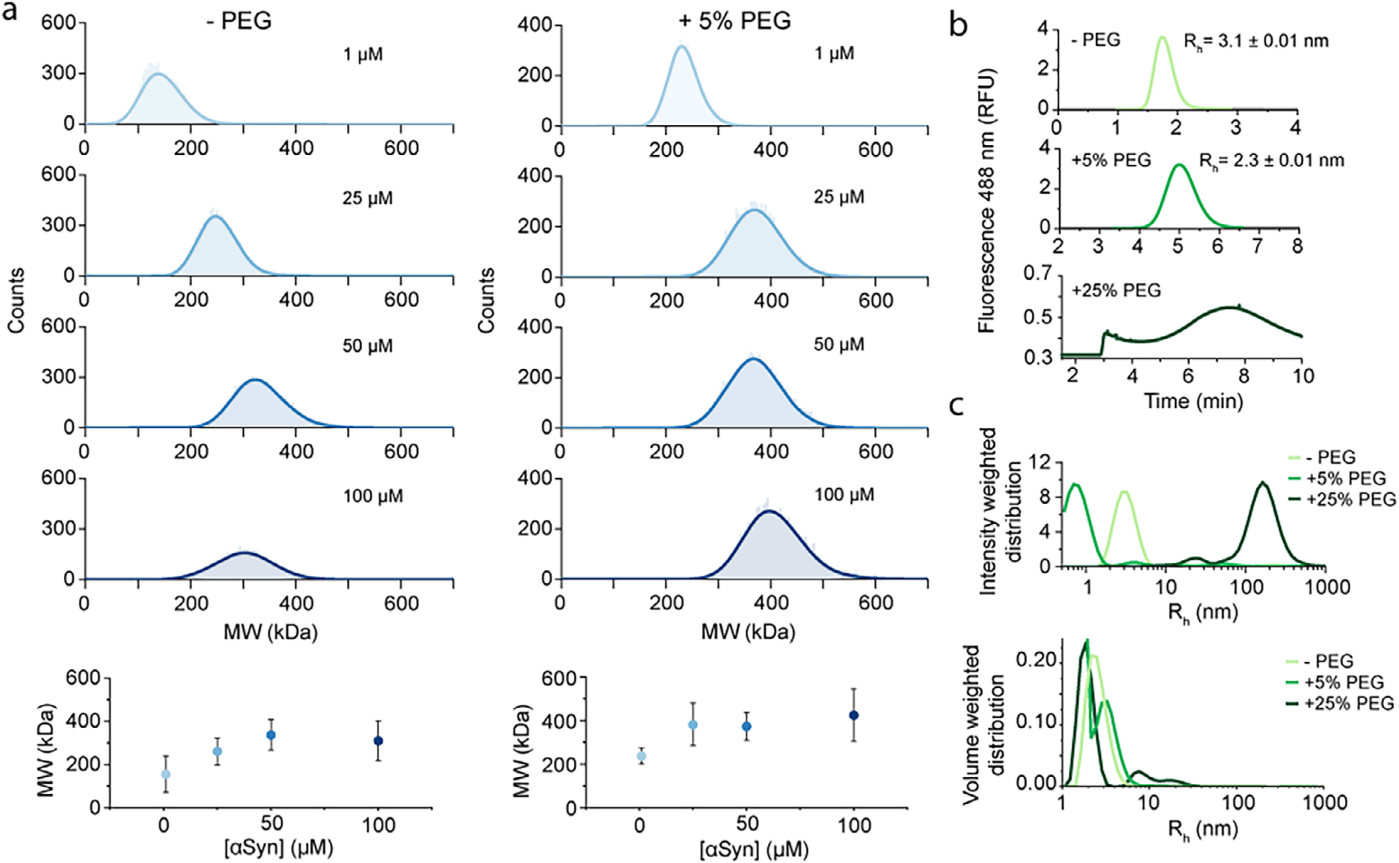
Detection of low‐salt α‐Syn assemblies with different experimental methods: (a) (top) Histograms of MP measurements of 1–100 µM α‐Syn in the absence (left) and presence (right) of 5% w/v PEG‐8000 showing formation of assemblies of ∼300–400 kDa and (bottom) mean MW_app_ and standard deviation as a function of protein concentration derived from gaussian fits of histograms (b) TDA of 100 µM α‐Syn with 10 µM 140C‐A488‐ α‐Syn without (top), with 5% (middle) and with 25% (bottom) PEG‐8000, showing the emergence of a non‐diffusive peak in the presence of high concentrations of PEG‐8000. (c) DLS‐derived size distribution of 100 µM α‐Syn at varying PEG concentrations (top) intensity weighted and (bottom) volume weighted. At 25% PEG‐8000, we find an additional peak at R_h_∼9 nm. All samples are prepared in 20 mM NaPi, pH 7.4. Results shown are representative data from at least three experimental repeats with similar results.

MP measurements are normally recommended to be performed in the low nM range of protein concentrations [34, 35]; well below the concentrations used in our experiments. Saturation of the surface with monomeric protein with molecular weight below the detection limit can lead to a significant reduction of the probability of binding, and hence detection, of larger assemblies. Furthermore, high protein concentration on the surface can result in overlapping signals, which could be falsely interpreted as larger assemblies [34]. To confirm that our observations are indeed caused by the presence of α‐Syn LSAs, we performed experiments under similar conditions and concentrations with bovine serum albumin (BSA) as a control (Figure S1a). This folded protein is known to assemble into small oligomers of defined MW, but in our conditions is not expected to form larger assemblies [36]. We observed an equilibrium shift between monomeric to dimeric and trimeric states, but no spurious signal suggesting higher‐order assembly was found to appear as a consequence of the increased protein concentration (Figure S1a). Furthermore, to address the potential effect of the glass surface, where the LSAs are observed in MP, we repeated our experiments with a variety of surface‐modified slides: APTES coated [37], plasma cleaned (hydrophilic), and fluorocarbon‐ (AQPL) coated (amphiphobic) [38]. The formation of the α‐Syn LSAs was observed in all cases (Figure S1b). The large assemblies found specifically on the AQPL coated slides were likely caused by the aggregation of α‐Syn in the presence of a hydrophobic surface‐water interface [39] (Figure S1b).

To further rule out the necessity of surfaces in the formation and observation of LSAs, we employed the FIDA platform to perform Taylor dispersion analysis (TDA) of the LSAs in solution. In TDA experiments, larger, non‐diffusive protein assemblies can be distinguished from the diffusive monomeric protein through transient incomplete separation in laminar flow [40, 41, 42]. To obtain a reliable signal, we added C‐terminally Alexa488 labeled α‐Syn (140C‐A488‐ α‐Syn) to the wild‐type (WT) α‐Syn (1 to 10 ratio of labeled vs non‐labeled). At concentrations and conditions used in our MP experiments, only one peak was detected, corresponding to monomeric α‐Syn (hydrodynamic radii R_h_ = 3.1 ± 0.01 nm and 2.3 ± 0.01 nm in the absence or presence of 5% PEG8000, respectively, Figure 1b, top and middle). To probe whether the absence of larger species in these TDA experiments is simply due to the low probability of labelled protein incorporation, we repeated the experiment with a UV‐detectable F94W mutant of α‐Syn (Figure S1d). We observed similar results, with a skewed monomer peak due to significant adsorption of α‐Syn to the capillary walls under the low ionic strength conditions.

MP is a single particle technique that can detect oligomers/clusters even at very low volume fractions, in particular if the molecular weight of the excess monomer is below the detection limit. TDA, on the other hand, has no lower detection limit for the size of species, but provides only time‐averaged transport properties that include mass‐transfer effects (e.g., dissolution). Consequently, concentrations required for reliable resolution are higher compared to MP. The difference between TDA and MP results, and the given lower detection limit for the TDA instrument (0.1 nM, as indicated in the instrument specifications) indicate that the proportion of LSAs in the α‐Syn sample at equilibrium is very low (<< 1%) with the sample being predominantly monomeric, also explaining why the LSAs have not been previously detected with other techniques such as NMR [43, 44, 45]. Since the MP data with 5% (w/v) PEG‐8000 showed no change in the MW_app_ of the clusters, we hypothesized addition of higher concentration of PEG‐8000 would allow us to promote LSAs formation sufficiently to render them detectable by TDA. Therefore, we carried out a series of TDA experiments at similar α‐Syn concentrations (130 µm), but in the presence of 25% instead of 5% (w/v) PEG‐8000. Interestingly, we observed a transient signal (t_arrival_ 3 min) relative to a diffusive peak corresponding to the α‐Syn monomer (Figure 1b, bottom), suggesting the presence of higher order species in the sample.

### 2.2 LSAs are Monodisperse and Distinct From Other Assembly States

While MP, TDA, and DLS confirmed the existence of α‐Syn clusters at low ionic strengths, none of these techniques could provide morphological, topological, or dimensional information on these assemblies. To address these limitations, we turned to TEM and AFM. TEM can provide details on 2D shape and diameter distributions of small molecular assemblies. For a comprehensive characterization of the LSAs size distribution, we imaged α‐Syn at three different concentrations (20, 50, and 200 µm), and in the presence of 5% (w/v) PEG‐8000, where we observed size‐limited LSAs in MP. All three samples showed highly monodisperse populations of nm sized, spherical α‐Syn assemblies, distinct from structures seen in control BSA micrographs (Figure 2a–c, Figure S2a). These LSAs showed a narrow diameter distribution with similar median values (resolution limit for negative‐stain TEM ∼2 nm [46]) for 20 µM (26 nm ± 5 nm) to 50 µM (28 nm ± 6 nm), and 200 µm protein concentration (29 nm ± 4 nm) (Figure 2b). Furthermore, while these LSAs showed occasional clustering, we notably could not detect any evidence of them being capable of fusion, unlike high‐salt assemblies. We note that the clustering of these LSAs could be a drying artefact during TEM grid preparation (see methods). Lastly, we acquired images of 100 µm α‐Syn at three different PEG concentrations (5%, 10%, 25%) to confirm that the addition of PEG did not substantially change the morphology of the LSAs, as has been shown previously for other α‐Syn assemblies [8, 47]. While we observe larger assemblies, consistent with our TDA results, spherical LSAs with similar average diameters remain the dominant species for all three PEG concentrations (Figure S2b–d).

**FIGURE 2:**
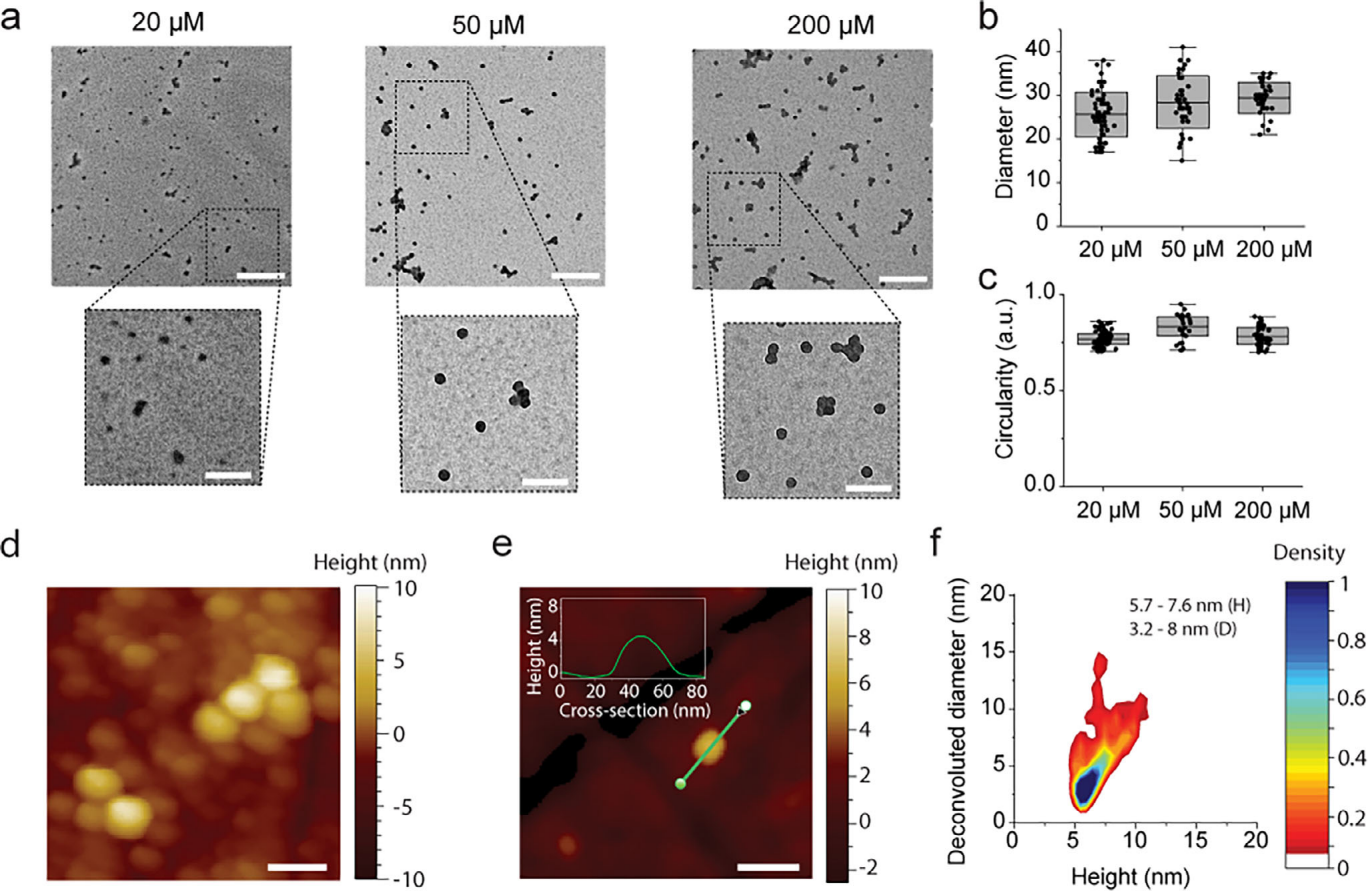
Microscopic detection and size analysis of LSAs: (a) Representative TEM images showing the presence of monodispersed LSAs in samples containing 20, 50, and 200 µM α‐Syn in the presence of 5% w/v PEG‐8000. Scale bar: 500 nm, inset 200 nm. (b, c) Box‐whisker plot showing the diameter (b) and circularity (c) distribution of the LSAs with standard deviation from n = 64 (20 µM), n = 39 (50 µM), and n = 34 (200 µM). (d, e) High‐resolution AFM maps of (c) multiple LSAs and (d) a single LSA formed at 50 µM protein concentration, in the presence of 10% w/v PEG‐8000. Image size: 250 × 250 nm. Image resolution: 2 nm/pixel. Scale bar: 50 nm. An example cross‐section versus height profile of the single LSA is shown in the inset. (f) 2D normalized kernel density plots show correlation between deconvoluted diameter and height of LSAs. N = 231. All samples are prepared in 20 mM NaPi, pH 7.4.

Next, we employed AFM to obtain three‐dimensional and surface topology information of the LSAs. We performed our experiments at 50 µM α‐Syn, where we could reliably detect spatially isolated, single LSAs in TEM. We also increased the PEG concentration to 10% in order to increase the volume fraction of LSAs to obtain meaningful size distributions, as AFM high‐resolution maps are typically acquired with a smaller field of view compared to conventional EM. We observed spherical assemblies (Figure 2d) with no obvious surface features, which were absent in control samples (50 µM α‐Syn in the absence of PEG‐8000, PEG‐8000 alone) (Figure S3a–h). Analysis of individual assemblies from a sparsely populated region (Figure 2e) revealed a narrow size distribution with dimensions of 5.7–7.6 nm x 3.2–8 nm in the first to third quartile, with an estimated median volume (V_h_) of 700 nm^3^ (Figure 2 and Figure S3i,j for analysis details). Our AFM measurements were performed in air on a dried sample, which does not necessarily resemble native conditions. We observe volumes corresponding to a molecular weight in an interquartile range of 300–1400 kDa, with a median of 600 kDa, which is slightly higher than the values obtained from MP measurements. The further difference observed between TEM and AFM can be attributed to the former method measuring only a projection of the 3D diameter of the LSAs, while AFM directly measures their 3D shape. We also note that we observed a proportion of larger species (∼18% of the population) in AFM, likely corresponding to clusters of LSAs as observed in TEM.

Finally, we evaluated the observed zero‐salt AFM size distribution in light of our TDA results obtained under identical conditions (Figure 1b). With the diffusion coefficient of monomeric α‐Syn *D* ≈ 3.2 · 10^−12^ *m* ^2^/*s* in 25% (w/v) PEG‐8000, the monomer is predicted to be transported diffusively under the experimental conditions employed in our TDA experiments (Gaussian‐like distribution in t, peak at ∼7.5 min). LSAs (*r* ≈ 8 *nm*,  *D* ≈ 10^−12^ *m* ^2^/*s*) and higher order aggregates *D* ≪ 10^−12^ *m* ^2^/*s* will be detected earlier due to slower diffusion leading to a residual influence of original radial position in the capillary (see Supporting Information). These larger species will contribute to the first peak at *t* ≈ 3 min. This distinct diffusive behaviour confirms the presence of higher order aggregates in zero‐salt conditions.

### 2.3 LSAs have Micellar Characteristics

The monodispersity, finite size, and lack of fusion of the α‐Syn LSAs suggests a micellar assembly state, akin to other IDPs that have recently been shown to undergo such phase transitions at sub‐saturated concentrations [26, 48, 49, 50]. With its domain organization, α‐Syn presents a block architecture, suggesting that LSA formation could be driven by the burial of the hydrophobic core as seen in a core‐shell micelle organization. To test whether the micellar characteristics were linked to the charge‐block nature of the sequence of α‐Syn (Figure 3a), we performed MP measurements with α‐Syn positive charge removal variants [51]. We tested three amphipathic domain mutation variants with one (K6Q), three (KQ1 = K6/10/12Q) and five (KQ12 = K6/10/12/21/23Q) lysines removed (Figure 3b) as well as two NAC mutation variants with 3 (KQ56 = K58/60/80Q) and 4 (KQ67 = K80/96/97/102Q) lysines removed (Figure 3c). Additionally, we also measured nanoscale assembly formation by the Core variant of α‐Syn (residues 30–110) lacking the majority of the N‐ and C‐terminal domains [8]. The core variant failed to show detectable assemblies in MP and additional FIDA measurements did not show a non‐diffusive peak but rather spikes corresponding to “macroscopic” (i.e., micrometer‐sized) assemblies in the presence or absence of NaCl (Figure S4a,b). Strikingly, while the NAC charge variants showed little change relative to wild‐type α‐Syn (Figure 3c), we observed that the amphipathic domain variants produced assemblies distinctly different from that of the WT, progressively decreasing in MW_app_ with increasing number of lysine removal to an approximately twofold decrease in MW_app_ (∼200 kDa to ∼100 kDa) for KQ12 (Figure 3b,d). This indicates that assembly size upon charge removal is dependent on the sequence location, an effect that is less expected for a disordered, bulk condensate‐like assembly. Indeed, contrary to LSAs, macroscopic condensates formed from WT and KQ mutants 1, 12 and 67, showed no significant difference in morphology as well as dilute phase concentration (Figure S4c.d), suggesting that these mutations have little impact on bulk assemblies.

**FIGURE 3:**
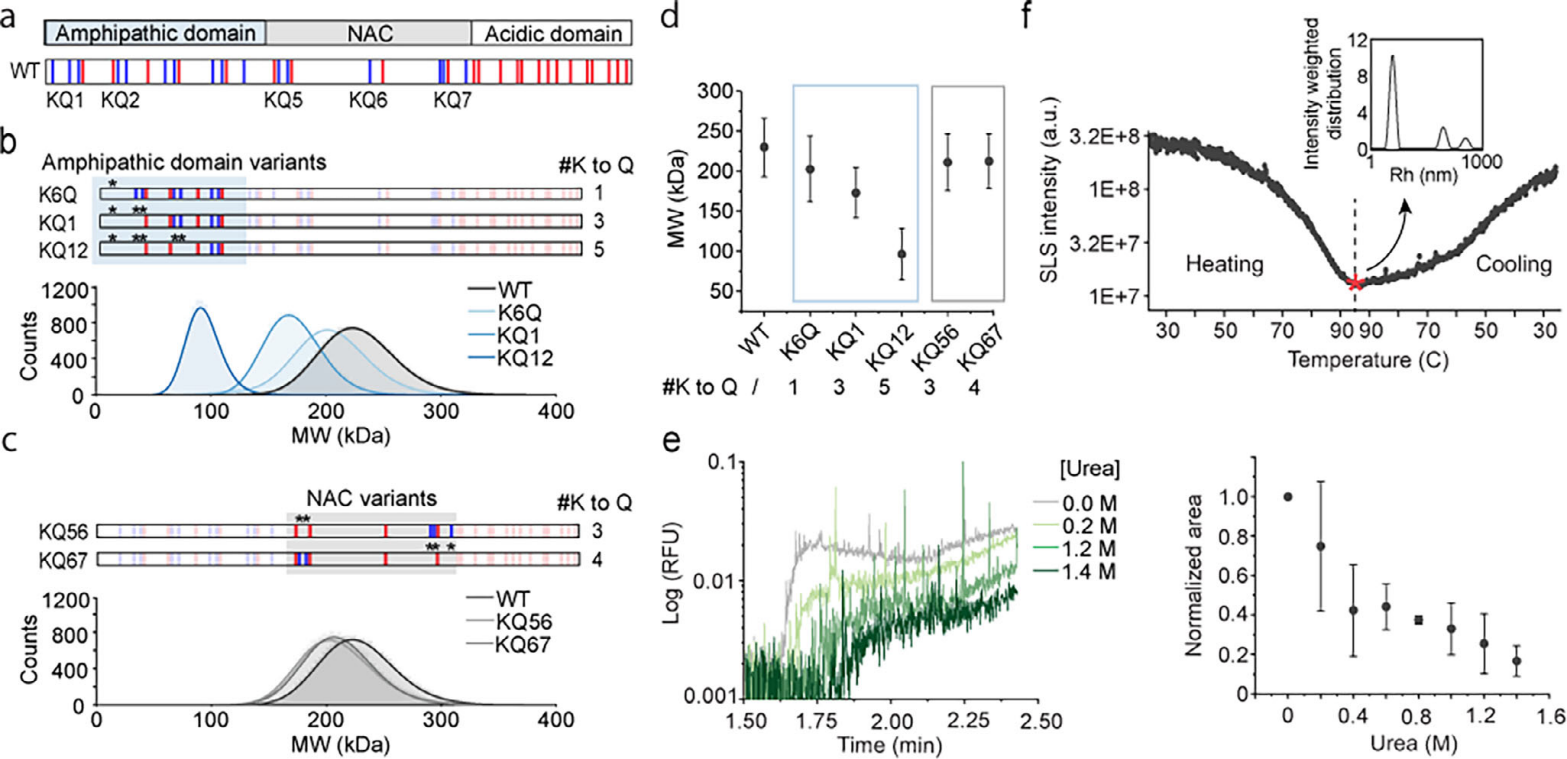
Stability measurements of α‐Syn LSAs: (a) Charge distribution for WT α‐Syn at pH 7.4 (negative in red, positive in blue) (b, c) MP measurements at 10 µM α‐Syn K to Q mutations for (b) amphipathic domain variants and (c) NAC variants. (d) Mean MW_app_ and standard deviation derived from gaussian fits of histograms (b and c) showing a negative dependence of MW_app_ with increasing numbers of K to Q mutations for amphipathic domain variants (light blue), while MW_app_ remained stable for NAC variants (grey). (e) (Left) TDA measurements of 200 µM α‐Syn+20% w/v PEG‐8000 in the presence of increasing concentrations of urea focused on the non‐diffusive transient signal corresponding to the LSAs. The signal progressively decreases as a function of urea concentration. (Right) The integrated area under the non‐diffusive transient peak plotted as a function of urea concentration with standard deviation from three experimental repeats. (f) Static light scattering (SLS) signal from LSAs formed at 100 µM α‐Syn + 20% w/v PEG‐8000 showing transition during heating and cooling cycles (25°C–95°C) indicative of reversible assembly. At 90°C, the presence of monomeric α‐Syn protein is confirmed from the DLS profiles showing R_h_∼3 nm (inset). All samples are prepared in 20 mM NaPi, pH 7.4. Results shown are representative data from at least three experimental repeats with similar results.

Urea titrations are typically used to probe the stability of higher order protein assemblies, such as amyloid fibrils. We therefore next probed urea dependent LSA stability using microfluidic transient incomplete separation [52], a method previously used to quantify the stability of α‐Syn fibrils in FIDA. Increasing concentrations of urea (0–1.6 m) were added to the buffer, and the non‐diffusive peak corresponding to the LSAs progressively disappeared (Figure 3e, left), demonstrating that these assemblies are substantially less stable than α‐Syn amyloid fibrils or oligomers (stable up to 4 M urea, Table 1) [52, 53].

**TABLE 1:** Experimental conditions used in TDA experiments.

Experiment Reference	Indicator	Analyte	Indicator mbar/s	Analyte mbar/s	Detection
Assembly detection (Figure 1b)	10 µM α‐Syn_A488+100 µM α‐Syn_WT	20 mM NaP buffer pH 7.5	75/20	400/200	488 nm
	10 µM α‐Syn_A488+100 µM α‐Syn_WT+5% w/v PEG8k	20 mM NaP buffer pH 7.5 + 5% w/v PEG8k	150/20	400/600	488 nm
	10 µM α‐Syn_A488+100 µM α‐Syn_WT+25% w/v PEG8k	20 mM NaP buffer pH 7.5 + 25% w/v PEG8k	2100/20	2800/1000	488 nm
Assembly detection (Figure 1d)	100 µM α‐Syn_F94W	20 mM NaP buffer pH 7.5	75/20	400/200	UV
	100 µM α‐Syn_F94W+5% w/v PEG8k	20 mM NaP buffer pH 7.5 + 5% w/v PEG8k	150/20	400/600	UV
	100 µM α‐Syn_F94W+25% w/v PEG8k	20 mM NaP buffer pH 7.5 + 25% w/v PEG8k	2100/20	2800/1000	UV
Urea dissolution (Figure 3e)	200 µM α‐Syn_WT + 20 nM α‐Syn_A488 + 2 0% (w/v) PEG8k	20 mM NaP buffer pH 7.5 + 20% w/v PEG8k+varying concentration of urea	900/10	3500/630	488

Finally, we probed the disassembly and reassembly of the LSAs using a temperature ramp while monitoring static light scattering (SLS) intensity. The LSAs displayed a broad transition to a fully monomeric state with a transition temperature (T_crit_) of ∼75°C (Figure 3f). This aligns well with the upper critical solution temperature behavior of micelles formed by amphiphilic surfactants [54]. Furthermore, cooling across the T_crit_ resulted in reappearance of higher‐order assemblies, indicating reversible disassembly and reformation of the LSAs.

### 2.4 LSAs are Observed Under a Restricted Set of Solution Conditions

The different assembly states of α‐Syn are dynamically highly interconnected and depending on solution conditions, protein concentration and incubation time can coexist, nucleate or convert into other assembly states (see details on assembly states in Table S1) [55, 56]. Here, we investigated the effect of incubation time and changes in ionic‐strength on the LSAs. To account for potential refractive index changes due to the addition of NaCl in our MP measurements, we measured our usual standards (IgG and BSA) at 0 and 200 mM NaCl and found no change in ratiometric contrast (Figure S5a), consistent with our earlier study [24]. The narrow size distribution observed in MP experiments at 0 mM NaCl (50 µM α‐Syn, 5% PEG‐8000, Figure 4a, top) shifts substantially toward higher molecular weight at 200 mM NaCl, indicating the formation of much larger assemblies, i.e., high‐salt nanoclusters ranging from 1–5 MDa (Figure 4b, top). Similarly, in TDA, increasing NaCl concentrations in the surrounding buffer to 200 mm resulted in the disappearance of the non‐diffusive peak observed in the absence of salt (Figure 4a, bottom), and the emergence of “spikes”, discrete peaks corresponding to the simultaneous entry of a large number of monomers into the detector window. The lower limiting radius of a spike‐forming condensate was estimated above as $r_{s,min}∼200nm,\;n_{agg}∼10^{6}$, equivalent to the smallest high‐salt nanoclusters. The largest spikes are estimated to occur in a size range r∼2.5μm based on mass‐on‐streamline calculations (see additional information in the Supporting Information). Spikes in the Taylorgrams therefore reliably indicate bulk condensate formation, while continuous transient signals may arise from micelles and/or sub‐200 nm condensate droplets. Next, we probed the ability of the LSAs to nucleate amyloid formation, as has been shown for assemblies formed under high ionic‐strength [24]. We measured ThT fluorescence at quiescent conditions (no active shaking) over 7 days. No fluorescence increase was detected in the absence of NaCl (Figure 4c). In contrast, the addition of 200 mM NaCl to the same sample, which triggers macroscopic condensate formation, resulted in a rapid increase in ThT fluorescence (Figure 4d). Although we find a steady increase of ThT fluorescence even after 7 days, stable dilute‐phase monomer concentrations of α‐Syn from spun‐down samples after 72 h (Figure S5b) indicate that the aggregation reaction has reached a plateau, and the increase in ThT signal is possibly due to sedimentation of aggregates in the well of the multiwell plate.

**FIGURE 4:**
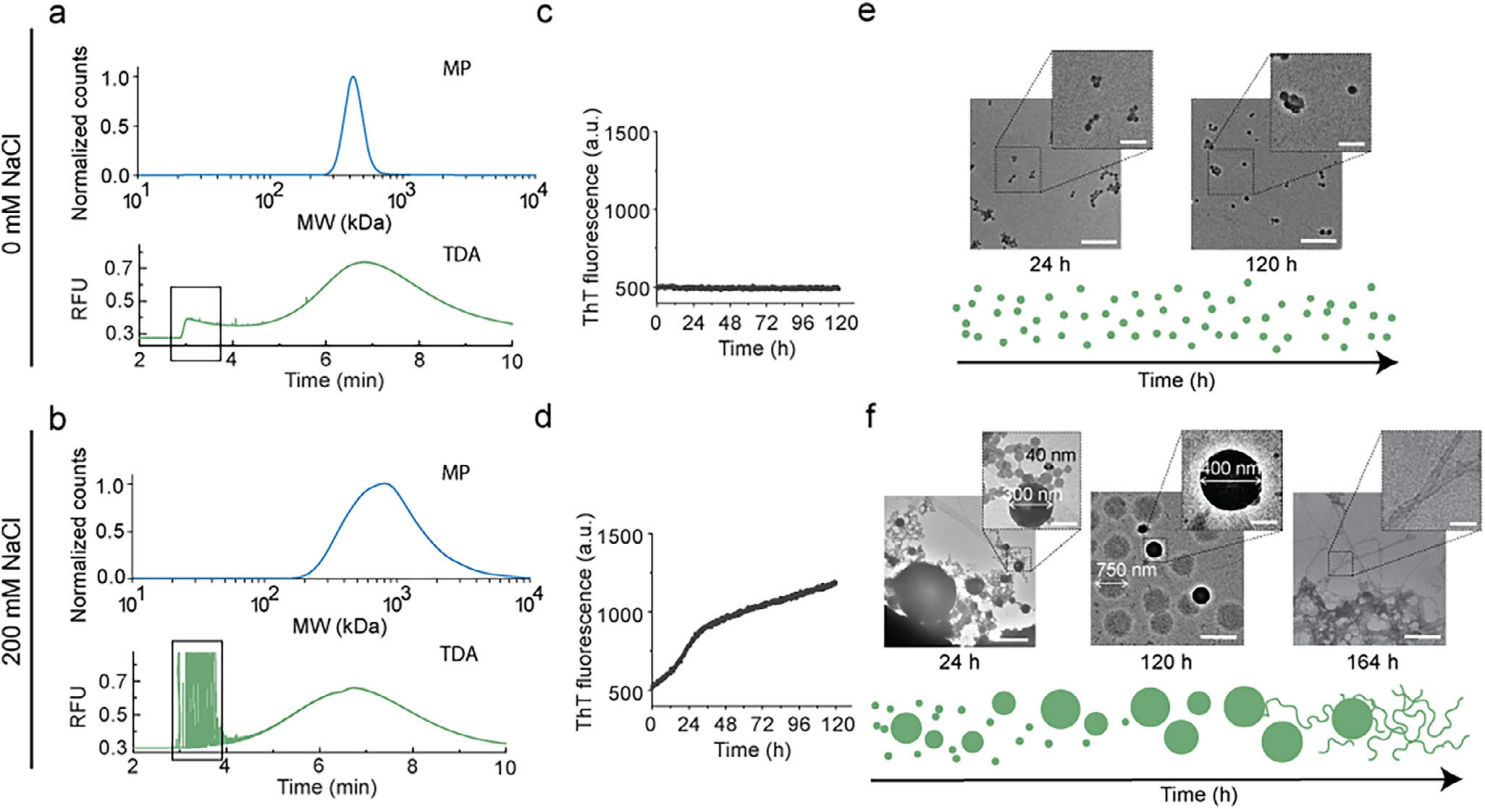
Aging of α‐Syn assemblies under different solution conditions. (a, b) LSAs (a) and condensates (b) formed in the absence or presence of 200 mM NaCl using MP (blue, no PEG) and TDA (green, 25% PEG‐8000). Detailed description of the criteria for emergence of a transient peak or spikes during TDA (black boxes) is provided in the supplementary note. (c, d) Kinetics of (c) LSAs or (d) condensates aging monitored under quiescent conditions at 25°C by Thioflavin T fluorescence. (e, f) Representative transmission electron micrographs of (e) LSAs and (f) condensates taken during their incubation at 25°C at indicated time‐points. Scale bar (e) 500 nm, inset 200 nm, (f) 1000 nm, inset 200 nm MP and TDA data are representative for duplicate experiments with similar results.

To investigate our ThT results further, we next collected TEM images of 0 and 200 mM NaCl samples after 24 h and 120 h of incubation at room temperature. In the absence of NaCl, LSAs are present in a narrow size distribution even after 120 h (Figure 4e). Importantly, in line with the results from ThT aggregation assay, LSAs did not nucleate any type of bulk assemblies on the time scales of this experiment. Contrarily, at 200 mM NaCl, we observed the emergence of bulk assemblies alongside smaller assemblies compatible in size with the LSAs. Notably, AFM images of LSAs at both conditions showed the presence of size limited assemblies, albeit with slightly higher volumes compared to the low‐PEG samples (*p* < 1 × 10^−07^, Figure S6). After 5 days, the LSAs had entirely disappeared in favor of phase‐separated condensates, which eventually led to the formation of some fibrils after 7 days (Figure 4f). This indicates that true phase‐separation of α‐Syn is energetically favorable in these conditions, with the micelle‐like LSAs disappearing over time due to monomer incorporation and coalescence upon collision. Together, these data suggest that LSAs are stable assembly states under quiescent low ionic strength conditions and neutral pH, where other α‐Syn assembly states are not energetically favored.

### 2.5 Distinction of α‐Syn From Other IDPs Undergoing Nanoscale Phase Separation

Nanoscale clustering at sub‐saturation has been shown for a variety of IDPs [19, 21, 22, 23, 24, 25, 26]. However, our observations suggest that, due to its distinct domain architecture, α‐Syn can form two different kinds of nanoscale assembly states under sub‐saturated concentrations: micelle‐ and condensate‐like, at low and high ionic strengths, respectively. We find that α‐Syn forms micelle‐like assemblies at conditions where its other assembly states (high‐salt nanoclusters/condensates) are unfavorable or kinetically inaccessible without additional perturbations (e.g., shaking, stirring [57]), whereas other IDPs show nanocluster formation under conditions where condensates are also favorable [19, 21, 22, 23, 24, 25, 26]. To investigate this difference in behavior in more detail, we performed MP measurements with two disordered proteins that have been shown to form spontaneous nano‐scale assemblies: TDP43‐LCD (274‐414) and Ddx4N1 (1‐236) [24, 25, 26]. Neither of these proteins have a clear block architecture, and they differ significantly in their amino acid composition. Ddx4N1 shows a high amount of charged residues, which are, unlike α‐Syn, not patterned but distributed along the entire sequence (Figure 5a,b). On the other hand, TDP43‐LCD is mostly dominated by hydrophobic and non‐polar residues (Figure 5a,b). As has been previously observed, condensate formation by Ddx4N1 is favorable at low NaCl concentrations [58, 59], while TDP43‐LCD condensate formation seems to be mostly independent of ionic strength at pH 7.5 [11].

**FIGURE 5:**
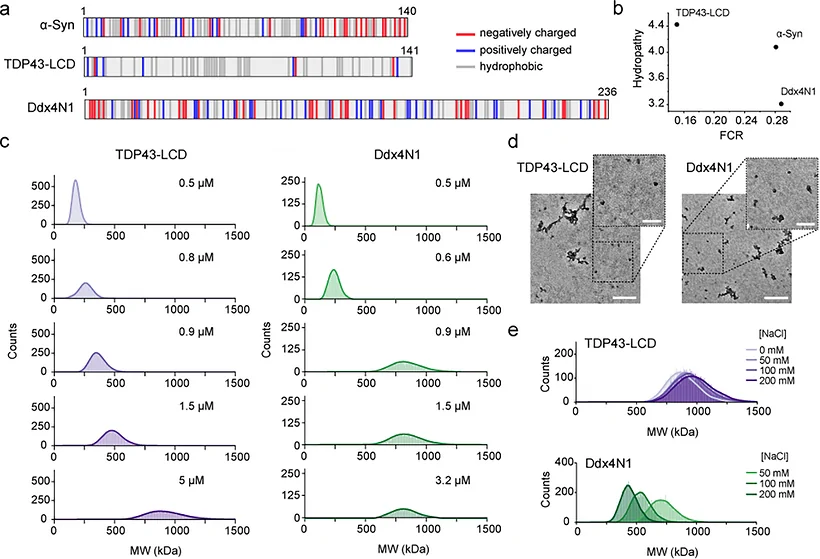
Nanocluster formation by TDP43‐LCD and Ddx4N1: (a) Charge distribution for α‐Syn, TDP43‐LCD and Ddx4N1 at pH 7.4 highlighting positive charged (blue), negatively charged (red) and hydrophobic residues (light grey). (b) Hydropathy versus fraction of charge per residue (FRC) plot generated using CIDER [60] for α‐Syn, TDP43‐LCD and Ddx4N1. (c) Histograms of MP measurements of TDP43‐LCD (0 mM NaCl) and Ddx4N1 (100 mM NaCl) at sub‐saturated concentrations showing formation of higher‐order assemblies for both proteins. (d) Representative TEM images confirming the presence of nanoscale clustering of both TDP43‐LCD and Ddx4N1. Scale bar: 200 nm, inset 100 nm. (e) Histograms of MP measurements of TDP43‐LCD (10 µM) and Ddx4N1 (10 µM) at increasing NaCl concentrations showing no change in the MW_app_ of the nanoclusters for TDP43‐LCD, but a decrease in the MW_app_ for Ddx4N1. TDP43‐LCD samples are prepared in 20 mM NaPi, pH 7.5. Ddx4N1 samples are prepared in 20 mM NaPi, pH 6.5.

MP measurements at subsaturated concentrations and low ionic strength showed the formation of nanoclusters for both proteins (100–900 kDa for TDP43‐LCD, and 900 kDa for Ddx4N1) (Figure 5c). This was subsequently verified with TEM imaging (Figure 5d), although due to their small size and less defined shape, no conclusions on the existence of a monodisperse, micelle‐like assembly state could be made. Notably, TDP43‐LCD showed a progressive increase in MW_app_ with increasing protein concentrations; but the MW_app_ of the Ddx4N1 nanoclusters stabilized and did not change beyond a maximum 900 kDa (Figure 5c,e). It is important to note that contrary to α‐Syn, the sequence and charge patterning of Ddx4N1 does suggest block‐copolymer‐like amphiphilicity. We therefore next investigated the effect of NaCl on these nanoclusters. Addition of NaCl did not change the MW_app_ of TDP43‐LCD nanoclusters but monotonically decreased the MW_app_ of Ddx4N1 nanoclusters. This meant that both proteins showed similar ionic strength dependence for these nanoclusters as for macroscopic condensate formation (Figure 5e). This is contrary to the behavior of α‐Syn, where addition of NaCl to the LSAs eventually led to the formation of MDa sized assemblies (Figure 3a [24]).

## 3 Discussion

In this work, we characterize the association of α‐Syn at sub‐saturated protein concentrations and low ionic strengths. Based on the known role of electrostatic interactions in nanocluster, condensate and fibril formation, it could have been expected that assembly or association of α‐Syn is highly disfavored at such conditions. However, α‐Syn assemblies at low ionic strengths have previously been observed in two independent studies using DLS — both in the absence and presence of PEG [61, 62]. In this work, we substantially extend this prior work by performing a systematic biophysical characterization of the WT and specific charge variants of α‐Syn. Using MP, we discovered low‐salt assemblies (LSAs) with characteristics that suggest a micellar architecture, albeit at a very low concentration. Increasing the volume fraction of the LSAs in PEG‐induced crowding conditions allowed us to further characterize these assemblies with complementary methods including TDA, TEM, AFM and ThT‐assays. The α‐Syn LSAs are monodisperse and maintain a constant size over a wide concentration range. This is in contrast to high‐salt nanoclusters and condensates, whose sizes increase with increasing protein concentration [24]. Furthermore, LSAs do not fuse upon contact and show no progression toward amyloid fibril formation. Lastly, we demonstrate that temperature induced disassembly of these clusters is reversible. Together, these properties hint toward these assemblies being distinct from previously described pre‐condensate clusters or percolated networks formed by disease‐associated IDPs [19, 21, 22, 23, 25, 26]. Based on the combination of our findings, we propose a micelle‐like conformation of the LSAs

Micelles are rare, sometimes functional assembly states for disordered proteins, such as caseins in milk [63] or, recently demonstrated, Matrin‐3 [64]. They are usually spherical or worm‐like shell‐core assemblies, are restricted in size to <100 nm and do not normally fuse [65, 66]. Building blocks of micelles are commonly defined by an amphiphilic domain architecture, to form a conformation where hydrophobic units are buried in the core while hydrophilic domains remain solvent facing. The specific shapes and sizes of micelles are governed by the shape of their individual building blocks, with bulky polar head groups and small hydrophobic tails favoring spherical micellar assemblies, such as e.g. in the classical case of the surfactant SDS [67]. The well‐defined geometry of a typical small surfactant contrasts strongly with the complex and ever‐changing structural ensemble of an intrinsically disordered protein. This is in particular true for α‐Syn, which has a highly variable geometry that strongly depends on the solution conditions [44, 45]. By exploring the dependence on protein, PEG, and NaCl concentration, we find that the association of α‐Syn into nanoscale clusters is defined by a delicate balance between electrostatic and non‐electrostatic interactions. While association is overall electrostatically unfavorable and aided by an increase in salt, the charged blocks of α‐Syn can still presumably interact favorably within a single molecule as well as across different molecules in a particular type of arrangement. This specific arrangement may only be accessible under a select set of solution conditions, where the attractive (local electrostatic, hydrophobic, Van Der Waals, and depletion interactions from molecular crowding) and the repulsive (global electrostatic) interactions maintain a fine balance.

Consistent with this picture, we find that modification or removal of the charged blocks modifies the ability of α‐Syn to adopt LSAs. Neutralization of lysine residues in the N‐terminal region markedly changed the micelle‐like assemblies, whereas equivalent perturbations in the hydrophobic NAC region had little effect. The isolated NAC fragment without the charged termini did not assemble into micelle‐like structures. Consistent with these observations we propose an ABC block copolymer‐like assembly akin to the one proposed for NEAT1_2RNP in paraspeckles [68, 69]. We suggest that the NAC region (block B) forms a buried hydrophobic core while the charged N‐ and C‐termini (Block A and C, respectively) remain solvent‐exposed, producing a hairpin‐like arrangement of α‐Syn monomers (Figure 6a). In such a configuration, the charged exterior stabilizes the interface with the solvent while also imposing electrostatic barriers to monomer addition. This conformation will likely only be adapted in assembled α‐Syn, contrary to other disordered proteins that lack a block‐like architecture, like the low complexity domain of FUS, where the soluble monomer can be found in a ‘paperclip’‐like conformation, under the same conditions where the formation of higher order assemblies like condensates and fibrils is also observed [70]. A hairpin‐like conformation has been described for α‐Syn‐dimers [47, 71], suggesting that dimers or small oligomeric species might be present under the solution conditions where we observe LSAs and which cannot be distinguished from monomer with the methods we apply in this study. Assuming a micelle‐like architecture, the stable size of this assembly is determined by the balance between free energy contributions from micellar growth (i.e., adding the hydrophobic NAC region of an additional molecule into the micellar core), energy decrease due to surface minimization; a decrease in conformational entropy of the blocks in the core, and the balance of local and global electrostatic interactions [65, 66]. While our data strongly suggests a micelle‐like assembly, coarse grained simulations or FRET experiments could provide valuable structural details to describe our LSAs in future studies, even though both computational as well as experimental structural studies of LSAs may be challenged by their low populations.

**FIGURE 6:**
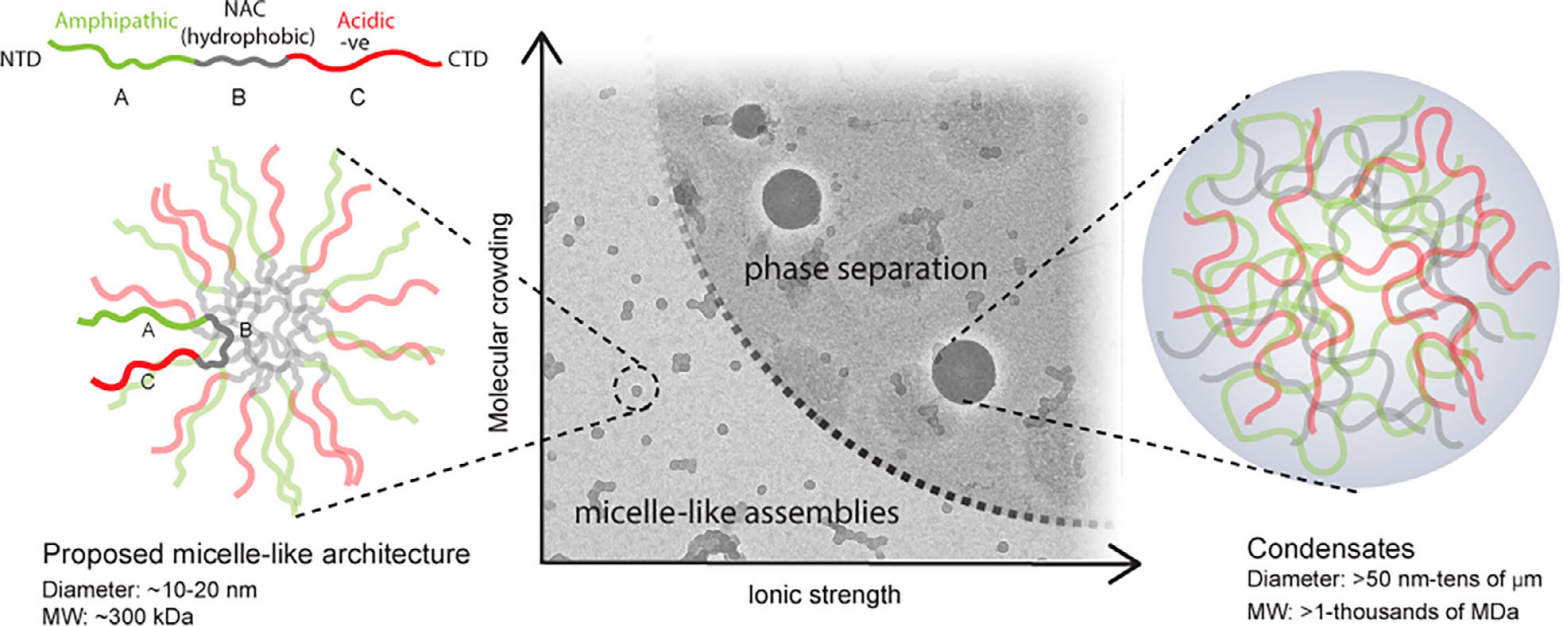
Proposed structural model for LSAs: Schematic showing a conceptual phase diagram highlighting the restricted solution conditions in which α‐Syn micelle‐like assemblies can be found. The proposed micelle‐like assemblies are (left) formed similar to ABC block copolymers, while condensates (right) are disordered.

Increasing the ionic strength of the solution shifts our LSAs toward high‐salt nanoclusters and condensates, both of which are likely to be more disordered, because screening of electrostatic interactions relaxes the relative orientational constraints of the molecules in the assembly. However, the micelle‐like LSAs appear to persist even in samples after addition of salt, suggesting they are at least kinetically stable. Cellular compartments like mitochondria, lysosomes, ER–mitochondria contact sites, and cytosol present distinct biochemical environments, affecting assembly states of α‐Syn. Behavioral differences of α‐Syn in these environments depend on several factors beyond ionic strength differences, including pH, crowding, and membrane composition [72, 73, 74, 75]. Furthermore, soluble monovalent anions in the cell are suspected to be much lower in concentrations (∼20 mm) than what is accepted as ‘physiological salt conditions’ (∼150 mM) [76]. It is therefore difficult to define an intracellular environment that is directly comparable to dilute in vitro conditions. However, the overlap of the ionic strength values in vivo with those that we tested in vitro suggests that nanoscale clusters of α‐Syn, including LSAs, are an assembly state also found in the cell.

Among other reported α‐Syn assemblies, the class of ∼30‐mer oligomers [27] has some resemblance to the micelles discussed in the present work, in terms of assembly number and size. However, these oligomers form predominantly during lyophilization [77], are not dynamic, and have a thermodynamic stability similar or higher to that of amyloid fibrils [53, 78]. The dynamic and reversible (after thermal denaturation) nature of the micelles described here clearly distinguishes them from any of the oligomeric states of defined size reported to‐date [27, 53, 78, 79, 80, 81, 82, 83], even though they may be precursors to some of the latter, e.g. when α‐Syn solutions are lyophilised at low ionic strength.

We also tested whether two other proteins previously reported to form nanoclusters, TDP43‐LCD and Ddx4N1 [24, 25, 26], are able to form micelle‐like assemblies. Among the two, we find evidence for Ddx4N1 micelles, i.e., dynamic assemblies the size of which is concentration‐independent, under a specific set of conditions. The formation of macroscopic condensates of α‐Syn and Ddx4N1 have the opposite dependence on ionic strength: An increase in salt concentration leads to a decrease in Ddx4N1 phase separation [59]. Ddx4N1 assemblies are destabilized at high ionic strength, in contrast to the non‐monotonic assembly behavior of α‐Syn, which forms disordered clusters and micelles at high and low ionic strengths, respectively. Despite this difference, the possibility of forming structures of well‐defined size that do not grow further upon the addition of more protein appears to be a general feature that they share. This finding suggests that the geometries and hydropathicity/charge patterns of IDPs are often sufficiently variable and flexible to comprise structures compatible with the formation of micelles [26, 64].

In summary, we believe this study presents strong evidence for an alternative and to‐date poorly characterized assembly state of α‐Syn. We propose that α‐Syn populates at least two classes of subsaturated assemblies: high‐salt nanoclusters that can be thought of as nanoscale condensates and that facilitate amyloid fibril nucleation, and low‐salt micelle‐like assemblies that are metastable and do not readily convert into fibrils (Figure 6b). Micelle‐like assemblies of IDPs could be a class of states accessible to many of the proteins that are able to undergo LLPS and condensate formation. Such species bridge the gap between oligomers of well‐defined size, composition, and structure on the one hand and large, disordered biomolecular condensates on the other.

## 4 Materials and Methods

### 4.1 Information on Plasmids

The plasmid encoding the untagged TDP‐43 low‐complexity domain (TDP43‐LCD; residues 274–414) in the pET‐3a expression vector was kindly provided by Kalyani Sanagavarapu (Lund University, Sweden). The Ddx4N1 CtoA plasmid (Ddx4N1‐eMM9‐TEV‐His) was kindly provided from Rasmus K. Norrild and is described in Norrild et al. [42]. Genes encoding wild‐type α‐synuclein and its NAC variant were purchased from Twist Bioscience cloned in pET29b(+). Mutant variants K6Q, KQ1 (K6Q+K10Q+K12Q), KQ12 (K6Q+K10Q+K12Q+K21Q+K23Q), KQ56 (K58Q+K60Q+K80Q), KQ67 (K80Q+K96Q+K97Q+K102Q), and A140C were prepared using Golden Gate mutagenesis protocol described in our previous study [51].

### 4.2 Expression and Purification of Recombinant α‐Syn

*α‐Syn wild‐type and KQ12 and KQ67 variant*: Bacterial cultures were grown in LB (at 37°C) until OD_600_ reached 0.8, and protein expression was induced by addition of 1 mM IPTG. The culture was grown for 4 more hours, and cells were harvested by centrifugation (7000xg, 20 min, 4°C). The cell pellet from 1 L culture was dissolved in 20 mL, 10 mm Tris‐HCl, 1 mm ethylenediamine tetra acetic acid (EDTA), pH 8.0 with 1 mM phenylmethylsulfonyl fluoride (PMSF) supplemented with Benzonase nuclease (Merck). The cells were then lysed by sonication on ice (10s on, 30s off, 12 cycles, at 40% amplitude). The suspension was then heated at 80°C for 20 min to precipitate the heat sensitive, contaminant proteins. The contaminant proteins were removed by centrifugation (20 000 xg for 20 min, at 4°C). The supernatant containing α‐Syn was collected, and the protein was salted out with 4 mL saturated (NH_4_)_2_SO_4_ (per mL supernatant). The salted out α‐Syn was collected using centrifugation (20 000 xg for 20 min, at 4°C). The pellet was dissolved in 25 mm Tris‐HCl, pH 7.7, and 1 mm dithiothreitol (DTT), followed by dialysis against the same buffer for 18 h at 4°C to remove (NH_4_)_2_SO_4_. The solution was subsequently subjected to anion exchange chromatography (AEC) (HiTrap Q Hp 5 mL, GE healthcare, USA) followed by size exclusion chromatography (SEC) (HiLoad 16/600 Superdex 200 pg. column). Concentrations were measured using NanoDrop Lite (Thermo Scientific, USA), by using the theoretical molar extinction coefficient 5960 M^−1^cm^−1^. Purified protein was flash‐frozen in liquid nitrogen and stored at −80°C until further use.

*NAC α‐Syn variant*: The NAC α‐Syn variant (residues 30–110) was purified using an identical protocol. However, due to the absence of the negatively charged C‐terminal tail, this variant was eluted in the flow‐through of the AEC.

*140C‐α‐Syn variant*: The 140C‐α‐Syn variant (used for fluorophore labeling) was also expressed and purified in the same way—the only difference being addition of 1 mM DTT and 1 mM EDTA in all buffers to prevent intermolecular disulfide linkages.

### 4.3 Fluorophore Labeling of 140C‐α‐Syn

For FIDA experiments Alexa488/647‐140C‐α‐Syn were used as a fluorescence reporter molecule. First, 14 mg/mL purified 140C‐α‐Syn was subjected to a Superdex 200 increase, 10/300 GL column to remove DTT/EDTA. Alexa488/647 C‐5 maleimide was dissolved in DMSO at a concentration of ∼7 mM. 360 µM 140C‐α‐Syn and 3.6 mM dye concentration was used for the labeling reaction, which was performed at 25°C for 1 h. Free dye was removed using Superdex 200 increase, 10/300 GL column. The concentration of the labeled protein was measured from the absorbance values at 275 nm (protein) and 488 nm (label) with NanoDrop Lite (Thermo Scientific, USA).

### 4.4 Expression and Purification of Recombinant TDP‐43 Low Complexity Domain (TDP43‐LCD)

*E. coli* BL21 (DE3) cell cultures carrying the TDP43‐LCD plasmids were grown in autoinduction media overnight at 37°C with shaking at 125 rpm. Cells were harvested by centrifugation (6000 xg for 30 min at 4°C), and pellets were stored at −80°C until purification. Frozen cell pellets were resuspended in 20 mL cold lysis buffer (25 mM Tris, pH 8.5, 250 mM NaCl, 1 mM MgCl_2_) supplemented with Benzonase nuclease (Merck). The cells were lysed by sonication on ice (2 × 1 min, 10 s on/off cycles at 60% amplitude). The lysate was centrifuged at 20 000 ×g for 20 min at 4°C, and the supernatant was discarded. The pellet was subsequently washed twice by resuspension in lysis buffer followed by sonication and centrifugation under identical conditions. Inclusion bodies were solubilized by re‐suspending the washed pellet in 20 mL of lysis buffer supplemented with 8 M urea, followed by sonication as described above. The sample was centrifuged at 20 000 ×g for 20 min at 4°C to obtain a clear supernatant, after which additional 20 mL lysis buffer were added. Cation‐exchange chromatography was performed using a HiPrep CM‐Sepharose 16/10 column (Cytiva) equilibrated with buffer containing 10 mM Tris base, pH 8.5, 4 M urea. The flow‐through fraction containing TDP‐43 LCD was subsequently up‐concentrated by centrifugation in rounds of 4 min, 3000 xg, 4°C, and then further purified by size‐exclusion chromatography on a HiLoad Superdex 75 pg 16/600 column (Cytiva) equilibrated with 20 mM sodium phosphate buffer, pH 7.5, 2 M urea. TDP‐43 LCD fractions were pooled and concentrated using centrifugal filters with a 10 kDa molecular weight cutoff (Merck). Purified protein was flash‐frozen in liquid nitrogen and stored at −80°C until further use.

### 4.5 Expression and Purification Ddx4N1

Ddx4N1 was expressed in *E. coli* BL21 (DE3) and cell cultures were grown at 37°C with shaking at 125 rpm, followed by induction with 1 mM IPTG and over‐night growth at 18°C. Cells were harvested with centrifugation (6000 xg for 30 min at 4°C) and cell pellets were resuspended in 50 mL lysis buffer (50 mM NaPi pH 6.5, 500 mM NaCl) with 1 mM PMSF and supplemented with Benzonase nuclease (Merck)) per 1 L culture. The cells were lysed by sonication on ice (10 min, 10 s on/off cycles at 30% amplitude) followed by centrifugation at 20 000 ×g for 20 min at 4°C. The supernatant was incubated at 80°C for 20 min to remove heat‐sensitive protein contaminants. The precipitated proteins were removed by centrifugation (4000 xg for 30 min at 4°C) and the supernatant was filtered through at 0.45 nm filter. The cleared supernatant was supplemented with imidazole to a final concentration of 10 mM and run over 5 mL Ni‐beads equilibrated in wash buffer 1 (50 mM NaPi pH 6.5, 500 mM NaCl, 20 mM Imidazole). The Ni‐beads were washed with 20 CV wash buffer 2 (50 mM NaPi pH 6.5, 500 mM NaCl, 20 mM Imidazole, 3 M GdnHCl) followed by 20 CV wash buffer 1. The beads were incubated over‐night at room temperature (RT) rotating in 10 mL lysis buffer with CombiTEVp (purified in‐house) and 2 mM TCEP to cleave off the His‐tag. The flow‐through containing the His‐cleaved protein was collected and the beads washed with 15 mL lysis buffer, which was combined with the flow‐through. The combined elution was up‐concentrated using centrifugal filters with a 10 kDa molecular weight cutoff (Merck) followed by incubation at 80°C for 5 min. The protein solution was centrifuged (13,000 xg, 10 min, RT) to remove protein aggregates and the supernatant was supplemented with NaCl to a final concentration of 1 M. This solution was subjected to size‐exclusion chromatography with a HiLoad Superdex 75 pg 16/600 column (Cytiva) equilibrated with SEC buffer (10 mM NaPi pH 6.5, 500 mM NaCl). Protein‐containing fractions were pooled and up‐concentrated using filters with a 10 kDa molecular weight cutoff (Merck). Purified protein was flash‐frozen in liquid nitrogen and stored at −80°C until further use.

### 4.6 Mass Photometry Measurements

All mass photometry measurements were conducted using a Refeyn TwoMP mass photometer (Refeyn Ltd., UK). Rectangular glass coverslips (24 mm × 50 mm; Fisher Scientific) used for the measurements were cleaned by sequential sonication in acetone (5 min, 100% amplitude), 50% isopropanol in Milli‐Q (MQ) water, and MQ water alone, followed by drying under a nitrogen source. Self‐adhesive silicone well cassettes (Refeyn Ltd., UK) were mounted onto the cleaned coverslips, which were subsequently placed on the 100x oil‐immersion objective. Before starting the measurements, the instrument lasers were allowed to stabilize for approximately 30 min. All experiments were performed at 25°C. Mass acquisition was recorded for 120 s unless otherwise specified. Frames were collected using optimized auto‐exposure settings in regular acquisition mode with a binned pixel dimension of 128 × 34, corresponding to a field of view of 10.8 µm × 2.9 µm and a detection area of 18 µm^2^. For each experiment, mass calibration was performed using protein standards of known molecular weight, including thyroglobulin (TG; 330 kDa monomer), bovine serum albumin (BSA, 66 kDa monomer and 130 kDa dimer), and immunoglobulin G (IgG; 145 kDa monomer and 290 kDa dimer). Standard proteins were diluted in the same buffer used for each experimental condition to a final concentration of 20 nm. Initial focusing and background subtraction were carried out using 9 µL of buffer, followed by addition of 1 µL of the protein standard. Measurements obtained from these standards were used to generate calibration curves converting ratiometric contrast into molecular weight. All subsequent measurements were calibrated using the curve obtained under the corresponding buffer conditions.

All datasets were acquired using AcquireMP software (v2.4.0) and analyzed using custom Python scripts (Python 3.12.7; NumPy, Pandas, Matplotlib, and SciPy libraries). For each dataset, histograms were generated using 500 bins over a MW range of 0–2000 kDa, corresponding to a bin width of 4 kDa. The same binning scheme was applied across all datasets to enable direct comparison of distribution shapes. Histograms were displayed as semi‐transparent filled distributions to visualize the underlying particle count distribution. To visualize the molecular weight distributions, Gaussian kernel density estimation (KDE) was applied using scipy.stats.gaussian_kde and used to estimate the probability density function by placing a Gaussian kernel at each data point and summing the contributions into a continuous curve. bw_method = ‘silverman’ was used to determine the KDE bandwidth factor, automatically selecting the smoothing parameter based on the data distribution.

*Mass photometry measurements with α‐Syn*: For measurements involving α‐Syn (WT, NAC, and mutants KQ12 and KQ67), 9 µL of 20 mm sodium phosphate buffer (pH 7.4) were deposited into a sample well and autofocus was performed using the droplet dilution function. Appropriate volumes of protein stock solutions prepared in the same buffer were then added to achieve the desired final concentrations to be tested. For experiments performed in the presence of NaCl, salt concentrations in both buffer and protein stock solutions were adjusted to ensure the intended final NaCl concentration after mixing. For measurements in the presence of 5% (w/v) PEG‐8000, appropriate volumes of PEG‐8000 solution prepared in 20 mm sodium phosphate buffer (pH 7.4) were added directly to the drop casted protein solution to reach the desired final concentrations of the reaction components. The solution was mixed with a pipette to allow homogeneous mixing of the PEG and measurements were initiated immediately afterward.

*Mass photometry measurements with TDP43‐LCD*: Mass photometry measurements for TDP43‐LCD were executed by first dispensing 9 µL of 20 mm sodium phosphate buffer (pH 7.5) into an individual well of the sample cassette. Protein samples, prepared as stock solutions in the same buffer, were subsequently added in appropriate volumes directly to the buffer droplet to reach the desired final concentrations. For NaCl concentration series measurements, 9 µL of the protein was added to the well and upon addition of 1 µL appropriate concentration of NaCl measurements were started.

*Mass photometry measurements with Ddx4N1*: Mass photometry measurements for Ddx4N1 were executed as for TDP‐43 LCD but in 20 mM NaPi pH 6.5.

### 4.7 Mass Photometry Measurements With Surface Modified Slides

*Plasma treated*: Glass slides were plasma cleaned (Plasma Cleaner PDC‐002‐CE). This was used to remove organic contaminants, and to render the glass surface hydrophilic and negatively charged.

*Aquapel treated*: Hydrophobic surfaces were prepared by Aquapel treatment, in which plasma‐cleaned coverslips were coated with a solution containing chloro(dimethyl)(3,3,4,4,5,5,6,6,7,7,8,8,8‐tridecafluorooctyl)silane (Merck) dissolved in Fluo‐Oil 40 (Emulseo).

*APTES treatment*: Positively charged surfaces were generated by (3‐Aminopropyl)triethoxysilane (APTES) treatment, a silanization procedure that functionalizes glass with amino groups. Briefly, the slides were sonicated in ethanol for 10 min, rinsed with MQ‐water and dried under a nitrogen source. Next, to activate the hydroxyl groups, the slides were immersed in 1 M NaOH for 15 min. Excess NaOH was washed with MQ‐water and the slides were dried under a nitrogen source. The slides were then immersed in 2% (v/v) APTES solution prepared in anhydrous ethanol for no longer than 15 min. The slides were washed with anhydrous ethanol, followed by MQ‐water and dried under a nitrogen source. Next, the slides were baked at 65°C for 2–3 h to allow efficient covalent bonding, and used immediately for the experiments.

### 4.8 Flow‐Induced Dispersion Analysis (FIDA) Measurements With α‐Syn

TDA measurements were carried out with non‐coated silica capillary (1 m length, inner diameter of 75 µm) at 25°C using FIDA1 instrument (Fida Biosystems ApS, Denmark). Each experimental run consisted of 2 wash steps (1 M sodium hydroxide, water), followed by filling the capillary by the analyte, injecting the sample (indicator) plug, and sample mobilization by the analyte solution and its detection. The experimental conditions and parameters used in different TDA experiments used in this study are provided in Table 1.

### 4.9 Dynamic (DLS) and Static (SLS) Light Scattering Experiments

DLS/SLS measurements were carried out using Prometheus Panta instrument (NanoTemper, Germany). Samples of 100 µM WT α‐Syn in the presence of 0%, 5%, and 25% (w/v) PEG‐8000 (20 mM NaP pH 7.5) were loaded into standard grade capillaries (NanoTemper, Germany) by capillary forces. Ten 5s acquisitions were collected for each sample, averaged, and the resulting correlation curves fitted to a size distribution model by the Panta Analysis software. The diffusion coefficients were corrected for viscosity and refractive index changes due to the presence of PEG to obtain reliable estimations of hydrodynamic radii. Buffer samples at varying concentrations of PEG without the protein were measured analogously to exclude any artifacts due to PEG scattering.

For the temperature reversibility experiments, scattering of a sample containing 100 µM α‐Syn in the presence of 25% (w/v) PEG‐8000 was measured during heating from 25°C to 95°C and subsequent cooling to 25°C at constant rate of 2°C/min.

**ThT fluorescence assay**: ThT aggregation kinetics with α‐Syn solutions were performed with 200 µm α‐Syn, 20% (w/v) PEG‐8000 in 20 mm sodium phosphate buffer, pH 7.4, and at 0 mm NaCl where only clusters are observed, and at 200 mm NaCl where the system transitions into forming macroscopic condensates. Equimolar (200 µM) ThT was added as an aggregation reporter to each sample. The samples were loaded onto low‐binding, clear bottom 96 well plates (Thermo Scientific, USA) and measurements were performed without shaking, at 25°C, using a fluorescence microplate reader FLUOstar Omega (BMG Labtech, Germany). The measurements were performed by exciting the samples at 440 nm and recording the fluorescence at 482 nm. The datapoints were recorded every 15 min for 7 days.

### 4.10 Atomic Force Microscopy (AFM)

*Sample preparation*: α‐Syn samples were prepared in 20 mM NaPi pH 6.5 with 50 µM α‐Syn plus 10% PEG in the absence and presence of 250 mM NaCl or with 100 µM α‐Syn plus 20% PEG. Additionally, we prepared two controls, 50 µM α‐Syn without PEG or NaCl (C_0_) or a buffer control with 10% PEG and 250 mM NaCl (S_0_). A volume of 3 µL was deposited on a fresh ZnS window (Crystran, UK) per each sample. To preserve sample integrity during the evaporation of the solvent, the deposition was sped up by letting the sample dry under vacuum. After checking for the complete evaporation of the solvent, the samples were rinsed three times with 1 mL of ultrapure water.

*Measurements*: AFM measurements of all the samples, excluded S_0_, were performed with an NX10 AFM (Park systems, South Korea) operating in non‐contact mode. PPP‐NCHR probes with a 42 N/m spring constant and ∼ 10 nm nominal tip radius were used for all the measurements. 2×2 µm^2^ images were collected with 2 nm/pix resolution and a 0.2 Hz scan rate. A free amplitude of 20 nm was imposed along with ∼ 16 nm amplitude set point for all images. S_0_ was imaged with a Multimode VIII AFM (Bruker, USA) operating in Scanasyst mode. A Scanasyst‐Air probe with 0.4 N/m spring constant ∼ 2 nm nominal tip radius was used. 2 × 2 µm^2^ images were collected with 1 nm/pix resolution and a 0.2 Hz scan rate.

*Analysis of AFM images*: AFM image analysis has been carried out with the software MountainsSPIP (Digital Surf, France). All the images were processed by applying a 0^th^ order line by line polynomial correction. To extract single‐molecule profiles from the 0.25 × 0.25 µm^2^ zoomed‐in images, a 7 × 7 median filter have been first applied. Particle diameter, height, and volume have been extracted through a height‐based threshold detection method. Specifically, a 4 nm height lower filter was set to ensure identification of particles above the substrate roughness (RMS = 1.5 ± 0.3 nm). To avoid artifacts, all the particles at the edges of the AFM images were discarded. Moreover, particles with area ≤ 75 nm^2^, volume ≤ 200 nm^3^ and roundness ≤0.6 were excluded. This further combination of filters allowed to exclude α‐synuclein species with low molecular weight (≤ 75 kDa; ∼ 5 proteins), particles smaller than the actual volume sensitivity, as well as amorphous aggregates. Statistical analysis (ANOVA with Tukey test) was performed on the logarithm of the volume data to obtain a normal distribution.

### 4.11 Transmission Electron Microscopy (TEM)

A 5 µL drop of each sample solution was applied to 400‐mesh carbon‐coated copper grids for 5 min. Excess sample was removed by gentle blotting with filter paper, and the grids were washed with a drop of Milli‐Q (MQ) water. Negative staining was performed twice using a 2 µL drop of 2% (w/v) uranyl acetate for 30 s per staining step. Following staining, the grids were washed with a drop of MQ water to remove residual stain and allowed to air‐dry at room temperature.

Samples were imaged using a Tecnai T20 G2 transmission electron microscope (FEI, USA) operated at 200 kV. Images were recorded using a TVIPS XF416 CCD 4K camera controlled by TVIPS EMplify software (version 0.6.23).

TEM images were processed using ImageJ by applying a Gaussian blur with σ = 2 nm, auto threshold “Triangle”, circularity threshold 0.7 to 1 and area threshold 200–1200 nm^2^. Circularity was given as the ratio of 4 pi times the area to the square of the perimeter.

## Author Contributions

S.H., S.R., and A.K.B. conceptualized the study, designed experiments, and wrote the manuscript. S.H., S.R., and F.S. performed and analyzed mass photometry experiments. Gi.N. and F.S.R. collected and analyzed AFM images. S.H., T.O.M., and Ge.N. collected and analyzed TEM images. S.R. and A.K. performed and analyzed TDA, DLS, SLS, and ThT experiments with the help of E.A. and D.J.O. T.O.M. analyzed TDA data for particle size distribution estimation. S.H., F.S., and A.K. expressed and purified proteins used in this study. All authors contributed to the finalization of the manuscript.

## Funding

This work is supported by ERC CoG (101088163 EMMA) to A.K.B., Lundbeck Foundation (Grant number 116392) to S.R., Horizon MSCA Individual Postdoctoral Fellowship (Grant number 101106115) to A.K, Lundbeck Foundation Postdoctoral Fellowship (Grant number R449‐2023‐1527) to S.H., Dutch Sector Plan β to F.S.R., Green DFF (Grant number 113737) to G.N., Novo Nordisk Foundation (Grant number NNF21OC0065495) and Lundbeck Experiment Grant (Grant number R400‐2022‐911) to T.O.M.

## Conflicts of Interest

The authors declare no conflicts of interest.

## Supporting information




**Supporting File**: advs77300‐sup‐0001‐SuppMat.docx.

## Data Availability

The data underlying this study are openly available in the Zenodo repository a https://doi.org/10.5281/zenodo.21669828.
