# Supplementary material for: α‐Synuclein Forms Distinct Micelle‐Like Assemblies at Low Ionic Strengths
Source: Adv Sci (Weinh). 2026 Aug 21:e77300. Online ahead of print. doi: 10.1002/advs.77300 (PMC13496287; doi:10.1002/advs.77300)
Supplement: Supplementary file 1 [file ADVS-9999-e77300-s001.docx]

**Supplementary information**

**α-Synuclein forms distinct micelle-like assemblies at low ionic strengths**

Sophie Hertel^1,#^, Soumik Ray^1,#^, Federica Saraceno^1^, Giacomo Nisini^2,3^, Georgia Nasi^1^, Thomas Oliver Mason^1^, Antonin Kunka^1^, Effrosyni Agrimaki^1^_,_ Dannie Juul Olesen^1^, Francesco Simone Ruggeri^2,*^, and Alexander K. Buell^1,*^.

^1^Department of Biotechnology and Biomedicine, Technical University of Denmark, Søltofts Plads, Building 227, 2800, Kgs. Lyngby, Denmark.

^2^Physical Chemistry and Soft Matter, Wageningen University and Research, Wageningen. The Netherlands.

^3^Laboratory of Organic Chemistry, Wageningen University and Research, Wageningen. The Netherlands.

#Co-first authorship.

*Correspondence:

Prof. Alexander K. Buell (alebu@dtu.dk), Department of Biotechnology and Biomedicine, Technical University of Denmark, Søltofts Plads, Building 227, 2800, Kgs. Lyngby, Denmark.

Associate Prof. Francesco Simone Ruggeri (simone.ruggeri@wur.nl), Physical Chemistry and Soft Matter, Wageningen University and Research, Wageningen. The Netherlands.

Supplementary Figures	1-7

Supplementary Table	8-9

Supplementary Note	10-11

References	11-12

**Supplementary Figures**

**Supplementary Figure 1**: a. Mass photometry (MP) measurement of bovine serum albumin (BSA) showing the presence of monomers (~66 kDa), dimers (~132 kDa) and trimers (~192 kDa) but no higher-order assemblies. b. MP measurements from Main Figure 1a repeated on APTES, AQPL and plasma treated slides. c) MP measurement of α-Syn at 10% PEG-8000PEG. Note that only a small number of assemblies are detected, likely because of surface passivation through PEG. d. Taylor dispersion analysis (TDA) of 100 µM α-Syn F94W mutant at increasing PEG concentrations using UV detection. Assemblies can only be observed as a transient shoulder at 25% PEG-8000. The peak corresponding to the diffusive species (monomer) is skewed (0 and 5% PEG-8000) or diminished (25% PEG-8000) due to the large degree of sticking to capillary walls. All samples are prepared in 20 mM NaPi, pH 7.4.

**Supplementary Figure 2**: a. TEM images of BSA showing precipitated protein without obvious high-order structure. Scale bar: 500 nm. b. Representative TEM images of 50 µM α-Syn at 10 % and 25 % PEG. Scale bar 500 nm, inset 200 nm. c and d. Size (c) and circularity (d) distribution of samples in b, showing that the addition of PEG does not have a significant effect on the characteristics of the LSAs. All samples are prepared in 20 mM NaPi, pH 7.4.

**Supplementary Figure 3:** a-d. Characterization of ZnS roughness after deposition of a control sample containing 0 µM protein concentration, 10% PEG-8000, and 250 mM NaCl. Root-Mean-Squared (RMS) = 1.5 ± 0.3 nm. Image size: 2x2 µm. Image resolution: 1 nm/pixel. e-h. Characterization of ZnS roughness after deposition of a control sample containing 50 µM protein concentration, 0% PEG, and 0 mM NaCl. RMS = 2.4 ± 0.3 nm. Image size: 2x2 µm. Image resolution: 2 nm/pixel. i and jRepresentative AFM high resolution map of α-synuclein nanoclusters formed at 50 µM protein concentration, 10% PEG-8000, and 250 mM NaCl. b. Corresponding AFM high resolution map where only the pixels belonging to the nanoclusters have been kept. All the white pixels outside nanocluster boundaries are void pixels. All the particles with maximum height ≥ 4 nm (2.5x ZnS roughness) are first detected. Then, to exclude spurious molecular species, further filters on area (A ≥ 75 nm^2^), volume (V ≥ 200 nm^3^), and roundness (r ≥ 0.6), have been used. The morphological parameters of the selected particles only have been analyzed. Image size: 2x2 µm. Image resolution: 2 nm/pixel. All samples are prepared in 20 mM NaPi, pH 7.4.

**Supplementary Figure 4:** a. MP measurements of 50 µM α-Syn NAC showing the absence of LSAs at both NaCl concentrations. b. Taylor dispersion analysis (TDA) of 90 µM α-Syn NAC with 20 nM 105C-A488- α-Syn with 25% PEG-8000 showing spikes corresponding to macroscopic assemblies across all NaCl concentrations. c. Representative microcopy images of α-Syn wild-type and mutant condensates. Scale bar: 10 µm. d. Dilute phase concentration of spun down condensate samples showing no significant difference between wild-type and mutants. All samples are prepared in 20 mM NaPi, pH 7.4, phase separation was induced with 300 mM NaCl and 25% PEG-8000 at 300 µM α-Syn

**Supplementary Figure 5: a.** MP measurements of BSA and immunoglobulin G (IgG) standards in the presence and absence of NaCl. While counts decrease in the presence of NaCl, no contrast shift is observed, suggesting a similar refractive index in the two conditions. b. Concentration of α-Syn in the soluble fraction of spun-down condensate samples from ThT-assay in main figure 4d at different time points. All samples are prepared in 20 mM NaPi, pH 7.4.

**Supplementary Figure 6: Representative** high-resolution AFM maps of (a and b) multiple low-salt α-Syn clusters and (c and d) a single α-Syn cluster. Image size: 250x250 nm. Image resolution: 2 nm/pixel. e and f Example cross-sections versus height profiles of the α-Syn LSAs from c and d. g and h 2D normalized kernel density plots show correlation between deconvoluted diameter and height of low-salt α-Syn nanoclusters. N = 231. All samples are prepared in 20 mM NaPi, pH 7.4, and measured at 25°C. Top row of figure samples were 50 µM protein in 10% w/v PEG-8000, 250 mM NaCl, bottom row figure samples were 100 µM protein in 20 % PEG-8000.

**Supplementary Tables**

**Supplementary Table 1:** Overview of α-Syn assembly states and their characteristics.

| **Assembly state** | **In vitro preparation** | **Ionic strength dependence** | **Critical concentration** | **Shape and size** | **Structure** | **Stability** |
| --- | --- | --- | --- | --- | --- | --- |
| Amyloid fibrils | Typically shaking/agitation, range of conditions (pH, additives), highly polymorphic^1^. | Aggregation rate increases with increasing ionic strength and decreasing pH. | low µM range | Elongated, nm-μm length, 6-20 nm width^2^. | Cross-β sheet core + disordered (fuzzy) coat | -26 - -43 kJ/mol^3^;  2-5M urea |
| Stable oligomers | Resuspension of lyophilized monomer, incubation of concentrated monomer at 37°C, filtration or size exclusion^4-9^. | Formation at neutral pH, low to medium ionic strength. | Stable at 1 μM | Ellipsoidal-cylindrical;  260-420 kDa, 18-34 monomers^4,9,10^ | Compact β sheet core, and disordered outer shell^9^. | Stable up to 8 M urea^10^. |
| Helical intermediate | Formed as a transient species during amyloid aggregation kinetics^11^. | Formed at low ionic strength,  20 mM Gly-NaOH, pH 7.4 | Transient species | Heterogeneous mixture of oligomeric species of various sizes | α-helical, with turns and β-sheet (FTIR), 5 nm height (AFM)^12^ | Transient |
| Macroscopic condensates | Spontaneous formation in presence of molecular crowder and in high salt buffer^13-15^. | Formed in high ionic strength buffers^13-15^. | 100 µM, under physiological buffer conditions, in presence of 15 % or more (w/v) PEG-8000^13-15^. | Spherical, micron scale entities. Detectable diameter range from 1-10 µm under a light microscope. | Unstructured at formation. Transition into amyloid fibril upon incubation^13-15^. | Stable up to 1M Urea at formation^15^. |
| Nanoscopic condensates (high-salt nanoclusters) | Spontaneous formation in presence of molecular crowder and in high salt buffer^13^. | Formed in high ionic strength buffers^13^. | 10 µM, under physiological buffer conditions, in presence of 5 % or more (w/v) PEG-8000 ^13^. | Spherical, nano scale entities. Detectable diameter range from 50-200 nm under TEM. | Possibly unstructured (condensate-like). | Stable up to 1M Urea at formation^13^. |
| Micellar clusters/LSAs(this paper). | Spontaneous formation in absence or in presence of molecular crowder and in low salt buffer. | Formed in low ionic strength buffers. | 1 µM, under physiological buffer conditions | Spherical, nano scale entities. Detectable diameter range from 10-50 nm under TEM/AFM.MP range: 300-400 kDa | Micellar. Our hypothesis: ABC block copolymer like architecture^16,17^. | Stable up to 1M Urea. |

**Supplementary Note**

**Size ranges observed in TDA experiments at high concentrations of PEG**

*Size criterion for advection-dominated transport- “transient peak”.*

First, we define size criteria for advective and diffusive behavior of species in Taylor dispersion under crowding conditions (25% w/v PEG-8000) in the absence of salt (Main Fig. 1b). Two peaks are observed, at t ≈ 180 and 408s. The first peak, characterized by sharp onset and slow decay, is referred to as a “transient” owing to the exponential decay of this contribution to the measured dispersion in time. It corresponds to the arrival times of species whose radial position in the capillary (and hence mean velocity) is not significantly altered by diffusion over the experimental timescale.

The typical criterion for the emergence of a transient peak is a limiting ratio of the diffusion coefficient ($D$) to the capillary radius ($R_{c}$), given as $\frac{Dt}{R_{c}^{2}}<0.6$^18^; where *t* is a characteristic arrival time. When such condition is satisfied, particles do not experience a “radially averaged” velocity (i.e., Taylor dispersion regime) and remain on flow streamlines. For our system (i.e., 25% PEG8000 solution with viscosity μ=27 mPa.s) we find the ratio at t ≈ 408s (corresponding to the average flow velocity) to fall below 0.6 for diffusion coefficients D < 2.1 10^-12^ m^2^/s, corresponding to species with Stokes-Einstein radius larger than 3.8 nm.

This demonstrates that any species larger than monomer ($D_{\alpha syn}\approx3.2\cdot{10}^{-12}m^{2}/s, r_{h}\approx2.5nm$,) will contribute a transient signal at the detector. The micelle-like LSAs, which are the smallest multimeric species observed in this study have $r_{h}\approx8$ nm and predicted Stokes-Einstein diffusion coefficient of $1\cdot{10}^{-12}m^{2}/s$ . LSAs will therefore be transported advectively and contribute to the transient signal.

*Size criterion for emergence of spikes.*

Here, we define criteria for splitting of the transient signal into discrete spikes as observed for αSyn under crowding conditions (25% w/v PEG-8000) in the presence of salt (>50 mM). Interval between detector frames is $\Delta t =0.02 s.$ The Poisson shot noise criterion determines signal continuity, where a frame-to-frame fractional variation is given by $\varepsilon=1/\sqrt{N}$ where $N$ is the number of species detected in each frame. $N$ is defined as $N=N_{mon}\left\langle n_{a}^{-1} \right\rangle$ where $N_{mon}$ is the total number of monomer molecules and $n_{a}$.the aggregation number (i.e., number of molecules in an assembly). For the lognormal distribution observed in the AFM experiments under these conditions (Main Fig. 4e), we find $\left\langle n_{a}^{-1} \right\rangle=exp(-\mu+\sigma^{2}/2)\approx12$, where μ and$\sigma^{2}$ are mean (3.584) and variance (2.232) of the natural logarithm of aggregation number. This gives $N\sim{10}^{7}$ independent particles from a $\sim{10}^{8}$ monomers/frame flux from an initial $\sim100\mu M$ plug. Based on these parameters, the AFM-observed distribution of LSAs is predicted to yield a smooth transient signal ($\varepsilon\approx3\cdot{10}^{-4}$), consistent with our TDA experiment (Figure 1b).

The lower limiting size of an aggregate that is recorded as a detector spike may be derived from the detector noise limit. Taking 4-times the empty-detector frame-to-frame signal standard deviation as an arbitrary threshold gives a signal value for spike $S_{spike}>{10}^{-3}RFU$. The peak diffusive signal from a 100 micromolar plug gives a detector concentration $\sim$1 micromolar which translates to $S_{pk}=0.4 RFU$. A spike above noise then corresponds to $O\sim{10}^{6}$ monomers, $r\sim200nm$assuming internal concentration 30mM.

A particle arriving at the detector that has “swept out” its axial cross-sectional area (all collisions with monomer resulting in addition) converts an entire cross-sectional circular domain of the initial diffusive monomer distribution into an aggregate travelling on the streamline. From the relation ${dr}/{dz}=\frac{v_{m}c_{n}}{4}$ , an initial isolated nucleus at the capillary center will increase in radius by $\sim2.5\mu m$ for $v_{\alpha syn}=55nm^{3}, c_{n}=6\cdot{10}^{22}/m^{3}$ if all collisions with monomer result in addition.

**References**

1	Sawaya, M. R., Hughes, M. P., Rodriguez, J. A., Riek, R. & Eisenberg, D. S. The expanding amyloid family: Structure, stability, function, and pathogenesis. *Cell* **184**, 4857-4873, doi:10.1016/j.cell.2021.08.013 (2021).

2	Ruggeri, F. S. *et al.* Identification and nanomechanical characterization of the fundamental single-strand protofilaments of amyloid α-synuclein fibrils. *Proceedings of the National Academy of Sciences* **115**, 7230-7235, doi:doi:10.1073/pnas.1721220115 (2018).

3	Fricke, C. *et al.* Thermodynamic stability modulates chaperone-mediated disaggregation of α-synuclein fibrils. *Chemical Science* **17**, 283-298, doi:10.1039/D5SC04927J (2026).

4	Chen, S. W. *et al.* Structural characterization of toxic oligomers that are kinetically trapped during α-synuclein fibril formation. *Proceedings of the National Academy of Sciences* **112**, E1994-E2003, doi:10.1073/pnas.1421204112 (2015).

5	Apetri, M. M., Maiti, N. C., Zagorski, M. G., Carey, P. R. & Anderson, V. E. Secondary structure of alpha-synuclein oligomers: characterization by raman and atomic force microscopy. *J Mol Biol* **355**, 63-71, doi:10.1016/j.jmb.2005.10.071 (2006).

6	Volles, M. J. *et al.* Vesicle Permeabilization by Protofibrillar α-Synuclein:  Implications for the Pathogenesis and Treatment of Parkinson's Disease. *Biochemistry* **40**, 7812-7819, doi:10.1021/bi0102398 (2001).

7	Paslawski, W., Mysling, S., Thomsen, K., Jørgensen, T. J. D. & Otzen, D. E. Co-existence of Two Different α-Synuclein Oligomers with Different Core Structures Determined by Hydrogen/Deuterium Exchange Mass Spectrometry. *Angewandte Chemie International Edition* **53**, 7560-7563, doi:10.1002/anie.201400491 (2014).

8	Lorenzen, N. *et al.* The Role of Stable α-Synuclein Oligomers in the Molecular Events Underlying Amyloid Formation. *Journal of the American Chemical Society* **136**, 3859-3868, doi:10.1021/ja411577t (2014).

9	Santos, J. *et al.* A Targetable N-Terminal Motif Orchestrates α-Synuclein Oligomer-to-Fibril Conversion. *Journal of the American Chemical Society* **146**, 12702-12711, doi:10.1021/jacs.4c02262 (2024).

10	Paslawski, W. *et al.* High Stability and Cooperative Unfolding of α-Synuclein Oligomers. *Biochemistry* **53**, 6252-6263, doi:10.1021/bi5007833 (2014).

11	Mehra, S. *et al.* α-Synuclein Aggregation Intermediates form Fibril Polymorphs with Distinct Prion-like Properties. *Journal of Molecular Biology* **434**, 167761, doi:[10.1016/j.jmb.2022.167761](https://doi.org/10.1016/j.jmb.2022.167761) (2022).

12	Ghosh, D. *et al.* Structure based aggregation studies reveal the presence of helix-rich intermediate during alpha-Synuclein aggregation. *Sci Rep* **5**, 9228, doi:10.1038/srep09228 (2015).

13	Ray, S. *et al.* Mass photometric detection and quantification of nanoscale α-synuclein phase separation. *Nature Chemistry*, doi:10.1038/s41557-023-01244-8 (2023).

14	Sawner, A. S. *et al.* Modulating α-Synuclein Liquid–Liquid Phase Separation. *Biochemistry*, doi:10.1021/acs.biochem.1c00434 (2021).

15	Ray, S. *et al.* Divergent effects of pathological α-synuclein truncations and mutations on phase separation. *bioRxiv*, 2024.2011.2018.624073, doi:10.1101/2024.11.18.624073 (2024).

16	Yamamoto, T., Yamazaki, T. & Hirose, T. Triblock copolymer micelle model of spherical paraspeckles. *Frontiers in Molecular Biosciences* **Volume 9 - 2022**, doi:10.3389/fmolb.2022.925058 (2022).

17	Yamazaki, T. *et al.* Paraspeckles are constructed as block copolymer micelles. *The EMBO Journal* **40**, EMBJ2020107270, doi:10.15252/embj.2020107270 (2021).

18	Alizadeh, A., Nieto de Castro, C. A. & Wakeham, W. A. The theory of the Taylor dispersion technique for liquid diffusivity measurements. *International Journal of Thermophysics* **1**, 243-284, doi:10.1007/BF00517126 (1980).
